# Dataset on cocoa production and climate change adaptation strategies in Ahafo Ano North District, Ghana

**DOI:** 10.1016/j.dib.2020.105275

**Published:** 2020-02-11

**Authors:** Abayomi Samuel Oyekale

**Affiliations:** Department of Agricultural Economics and Extension, North-West University, Mafikeng Campus, Mmabatho, 2735, South Africa

**Keywords:** Climate change, Adaptation methods, Vulnerability, Cocoa, Ghana

## Abstract

Sustainable cocoa production is susceptible to changes in some climatic parameters. This survey was carried out to understand the perceptions of cocoa farmers on climate change, its impacts on cocoa production and their adaptation methods. Stratified sampling method was used to select the farmers and data were collected with structured questionnaires. Stratification of the district was done based on existing seven administrative divisional offices which comprise of six area councils and one town council. Cocoa farmers were sampled within each stratum with sample size proportional to estimated number of farmers. During the survey, 378 cocoa farmers were interviewed from Abu-Bone (60), Anyinasuso (65), Biakoye (42), Kwasu-Abu (89), Subriso (35), Suponso (20) and Tepa (67). The dataset had been shared with this article and it is valuable for understanding the perceptions of cocoa farmers on climate change, cocoa production efficiency and determinants of climate change adaptation choices.

**Table:** Specifications Table

Subject	Agricultural and Environmental Sciences
Specific subject area	Climate Risk in Cocoa Production
Type of data	Table and charts
How data were acquired	Data were collected through sampling of cocoa farmers with structured questionnaire.
Data format	Raw and analyzed.
Parameters for data collection	Face to face interviews of cocoa farmers was carried out with structured questionnaires.
Description of data collection.	Data were collected using stratified sampling method. Stratification was done based on the seven existing administrative divisional offices. Samples were allocated to each stratum proportionately in accordance with estimated number of cocoa farmers. Interviews were successfully carried out among 378 cocoa farmers.
Data source location	Ahafo Ano North District in the Ashanti region of Ghana
Data accessibility	Dataset had been submitted with the article.

**Table:** 

**Value of the Data** • The dataset is useful because it can enhance our understanding of the different forms of climate change being perceived by cocoa farmers, the impacts they have on cocoa agriculture and the adaptation methods being used by the farmers; • This dataset can serve as quick reference for researchers and other stakeholders in the cocoa value chains in the design of policies for mitigating the impacts of climate change on cocoa production. • The dataset can be further explored to facilitate our understanding of the correlates of cocoa farmers' vulnerability to climate change; and • The data can also be used to evaluate the impact of climate adaptation on efficiency of cocoa production.

## 1 Data

The data file that is submitted with this paper comprise of information from 378 cocoa farmers. These farmers were interviewed from the six area councils and one town council of the Ahafo Ano North District of Ghana. The major objective of the survey was to understand the perceived impacts of climate change on cocoa production and the methods being used by the farmers to adapt. The issue of climate change is very relevant to cocoa agriculture given the sensitivity of many pests and diseases pathogens that affect cocoa production to changes in some climatic parameters [[1], [2], [3], [4]]. This dataset therefore offers some vital information to policy makers and stakeholders in the cocoa value chains on the pattern of changes already perceived by the farmers and their evaluation of current impacts on cocoa production.

**Fig. 1:**
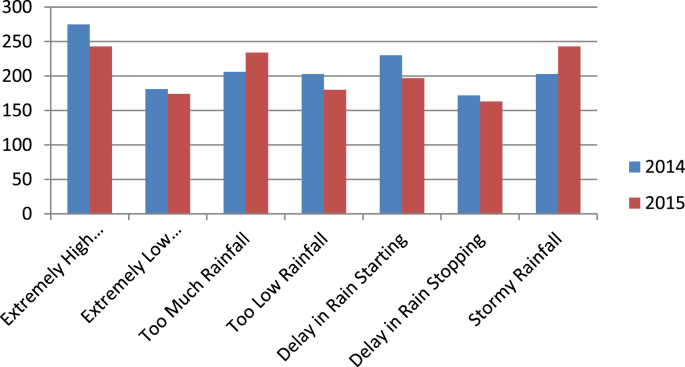
Frequency distribution data on perceived forms of climate change by cocoa farmers.

**Fig. 2:**
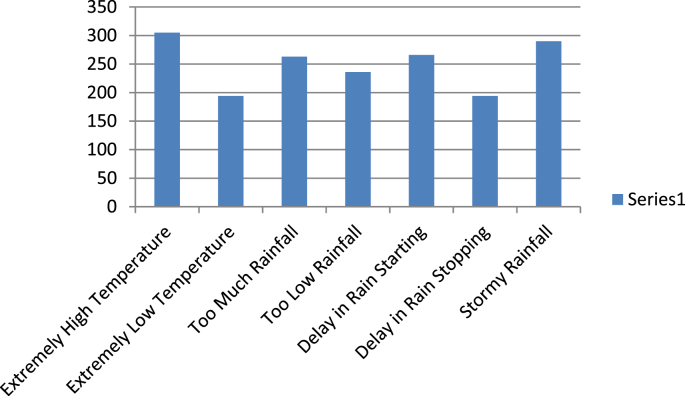
Frequency distribution data on the form of climate change that affects cocoa production.

The questionnaire which had been submitted as supplementary file sought information on cocoa farmers’ demographic information, input usage for cocoa production, perceived forms of climate change, impact of climate change on cocoa production and their adaptation methods. Table 1 shows the distribution of the respondents based on some selected demographic characteristics. The data show that average age of sampled cocoa farmers was 51,73 years. Average household size was 8.35 and average number of adult household members was 5.11. Average farming experience was 23.47 years while average cocoa farming experience was 18.80 years. The data presented as Fig. 1 show the distribution of the farmers based on perceived forms of climate change. The data show that majority of the farmers perceived extremely high temperature, too much rainfall, delay in rainfall commencement and stormy rainfall. The data in Fig. 2 show that among the perceived forms of climate change, extremely high temperature, stormy rainfall and delay in rainfall commencement were noted by the highest majority of the farmers to have very important influence on cocoa production.

**Table 1:** Selected demographic characteristics of cocoa farmers.

Socioeconomic factor	Average	Standard deviation
Age	51.73	12.69
Household size	8.35	4.73
Adult members (≥15 years)	5.11	3,29
Children less than 5 years	2.80	2.82
Cocoa farming experience	18.80	12.73
General farming experience	23.47	13.09

## 2 Experimental design, materials and methods

The data were collected in June 2015 from cocoa farmers in Ahafo Ano North District. This district is among the top cocoa growers the Ashanti region of Ghana. The survey was coordinated by the extension officers in the District who organized the enumerators’ trainings and facilitate contacts with the cocoa farmers. Preceding the survey was a training section to familiarize the enumerators with some terminologies in the questionnaire with a view to have a common understanding in guiding the farmers to answering some of the questions. The questionnaire comprises of three sections. Section one probed into the demographic characteristics of the farmers. Section two was on farm inputs being used for cocoa production and section three was on vulnerability to climate change and adaptation methods. The questionnaire was pretested with selected cocoa farmers before commencement of the survey. The sampling procedure utilized the existing administrative divisions of the distinct. The stratified random sampling method was used with each area/town council forming a stratum. Therefore, there were seven strata from where cocoa farmers were randomly sampled with sample size being proportional to size. A total of 378 cocoa farmers were successfully interviewed within the five days of the survey from Abu-Bone (60), Anyinasuso (65), Biakoye (42), Kwasu-Abu (89), Subriso (35), Suponso (20) and Tepa (67).
