# Supplementary material for: Dataset on cocoa production and climate change adaptation strategies in Ahafo Ano North District, Ghana
Source: Data Brief. 2020 Feb 11;29:105275. doi: 10.1016/j.dib.2020.105275 (PMC7033315; doi:10.1016/j.dib.2020.105275)
Supplement: Multimedia component 1 [file mmc1.docx]

**QUESTIONNAIRE ON**

**“CLIMATE CHANGE, OCCUPATIONAL SAFETY AND COCOA AGRICULTURE SOCIO-ECONOMIC SURVEY”**

Dear Respondent, Information required in the questionnaire will be used mainly for research purpose. Please, provide as accurate information as possible and we promise to ensure confidentiality. Thank you.

**Site Identification**

| Region Name | District Name | Village Name | Date of Interview | Interviewer’s Names |
| --- | --- | --- | --- | --- |
|  |  |  |  |  |

**A. HOUSEHOLD DEMOGRAPHIC INFORMATION**

1. Gender of the household head (Tick): Male Female

2. Age of the household head (years)

3. Does the head have any formal education? Yes No

4. If yes, what is the level of education? Primary Secondary Tertiary

5. How many years of cocoa farming experience do you possess?

6. How many of you are living and eating together?

7. Number of household members who are adults (>=15 years old)

8. Number of household members whoare <5 years old

9. How long ago did you start farming?

10. How long have you been growing cocoa?

11. Is cocoa the primary crop cultivated? Yes No

12. Is farming your primary occupation? Yes No

13. Estimate the monthly incomes realized by all household members from the following sources:

| Income Sources | Amount (Monthly) | Income Sources | Amount (Monthly) |
| --- | --- | --- | --- |
| Agricultural (crops) |  | Formal loans |  |
| Agricultural (livestock) |  | Non-farm business |  |
| Agricultural wage |  | Non-farm wage |  |
| Informal loans |  | Sales of assets |  |
| Begging |  | Remittances |  |

14. How many times did household head fall sick during last cocoa season?…………………………………

15. What is the nature of the major sickness?......................................................................................................

16. Did any other household member fall sick? Yes……………… No………………….

17. Did you miss regular cocoa spraying due to illness during the last cocoa season? Yes…… No……

18. Did you delay cocoa farm clearing due to illness last season? Yes……………….. No…………….

**B. COCOA FARMS’ INPUTS AND OUTPUTS**

19. How many cocoa farms do you have in this village?

20. Please, provide information on a particular cocoa pod producing farm.

| Type of farm ownership ** (A) | Land area (Acre) | Proportion of cocoa(%) | Year of cocoa tree planting | Year cocoa tree rehabilitation | Distance to the village (miles) |
| --- | --- | --- | --- | --- | --- |
|  |  |  |  |  |  |

1. Personal farm, B. Rented farm C. Lease farm D. Sharecropping E. others

21. Please, estimate the labour inputs (Amount Spent)

i. Hired labour (Ghanaian Cedis) ……………………………

ii Family labour (Ghanaian Cedis)……………………………..

22. Please, provide the number and value of other equipment used on cocoa farm

| Implements | Number | Estimated value (GC) | Implements | Number | Estimated value (GC) |
| --- | --- | --- | --- | --- | --- |
| Hoe |  |  | Spreading mats |  |  |
| Machete |  |  | Irrigation pump |  |  |
| Baskets |  |  | Rain boot |  |  |
| Sprayer |  |  | Go to hell |  |  |
|  |  |  | Others |  |  |

23. What are the quantities of other inputs used on cocoa farm during last season?

| Input | Type/Name | Quantity used | Cost of Qty Used | Adequate in supply or not? |
| --- | --- | --- | --- | --- |
| Fertilizer |  |  |  | Yes………….. No………... |
| Herbicide |  |  |  | Yes………….. No………... |
| Germicide |  |  |  | Yes………….. No………... |
| Pesticide |  |  |  | Yes………….. No………... |
| Others chemicals |  |  |  | Yes………….. No………... |

24. Please, provide some estimates of dried cocoa beans output during last season in 2014

| Cocoa output | Dried beans (kg) | Amount  (GC) | Losses to Black pod (kg) | Estimated value  (GC) | Losses to Rodents (kg) | Estimated value (GC) |
| --- | --- | --- | --- | --- | --- | --- |
| Jan-March |  |  |  |  |  |  |
| April - June |  |  |  |  |  |  |
| July – Sept |  |  |  |  |  |  |
| Oct- Dec |  |  |  |  |  |  |

**C. CLIMATE CHANGE, VULNERABILITY AND COCOA AGRICULTURE**

25. Have you noticed some changes in climate or weather parameters in this village? Yes….. No……..

26. If yes, what are the changes you have recently noticed and their rankings? (tick many)

| Observed climate changes | Last year | This year | Trend |
| --- | --- | --- | --- |
| Extremely high temperature | Yes….. No….. | Yes….. No….. | Up…. . Down….. Same….. |
| Extremely low temperature | Yes….. No….. | Yes….. No….. | Up…. . Down….. Same….. |
| Too much rainfall | Yes….. No….. | Yes….. No….. | Up…. . Down….. Same….. |
| Too low rainfall | Yes….. No….. | Yes….. No….. | Up…. . Down….. Same….. |
| Delay in rainfall commencement | Yes….. No….. | Yes….. No….. | Up…. . Down….. Same….. |
| Delay in rainfall stopping | Yes….. No….. | Yes….. No….. | Up…. . Down….. Same….. |
| Too stormy rainfall | Yes….. No….. | Yes….. No….. | Up…. . Down….. Same….. |
| Thicker cloud covers | Yes….. No….. | Yes….. No….. | Up…. . Down….. Same….. |

27. Do these changes impart negatively on households’ health? Yes………………. No…………………….

28. If yes, what disease is most rampart?............................................................................................................

29. What other climate-risk associated problems have you noticed? (tick as many as may be applicable)

| Observed climate changes | Last year | This year | Trend |
| --- | --- | --- | --- |
| Increase in the number of pests | Yes….. No….. | Yes….. No….. | Up…. . Down….. Same….. |
| Difficulties in weed control | Yes….. No….. | Yes….. No….. | Up…. . Down….. Same….. |
| Increase in malaria incidences | Yes….. No….. | Yes….. No….. | Up…. . Down….. Same….. |
| Scarcity of drinking water | Yes….. No….. | Yes….. No….. | Up…. . Down….. Same….. |
| Scarcity of cooking water | Yes….. No….. | Yes….. No….. | Up…. . Down….. Same….. |
| Higher incidence of pneumonia | Yes….. No….. | Yes….. No….. | Up…. . Down….. Same….. |
| Higher incidence of cholera | Yes….. No….. | Yes….. No….. | Up…. . Down….. Same….. |

30. How have some of these changes affected cocoa production and what is the trend?

| Observed climate changes | Last year | This year | Trend |
| --- | --- | --- | --- |
| Death of young cocoa plants | Yes….. No….. | Yes….. No….. | Up…. . Down….. Same….. |
| Cocoa plants falling off | Yes….. No….. | Yes….. No….. | Up…. . Down….. Same….. |
| Reduction in cocoa yield | Yes….. No….. | Yes….. No….. | Up…. . Down….. Same….. |
| Higher incidence of black pod disease | Yes….. No….. | Yes….. No….. | Up…. . Down….. Same….. |
| Higher incidence of wild fire | Yes….. No….. | Yes….. No….. | Up…. . Down….. Same….. |
| Inability to spay cocoa pods effectively | Yes….. No….. | Yes….. No….. | Up…. . Down….. Same….. |
| Inability to dry cocoa pods effectively | Yes….. No….. | Yes….. No….. | Up…. . Down….. Same….. |

Trend: 1- increasing 2- decreasing 3- the same

31a. Which of the following currently affects your cocoa production activities? (Tick for yes)

| Climatic changes variables | Response | Climatic changes variables | Response |
| --- | --- | --- | --- |
| Extremely high temperature | Yes….. No….. | Too low rainfall | Yes….. No….. |
| Extremely low temperature | Yes….. No….. | Delay in rainfall commencement | Yes….. No….. |
| Too much rainfall | Yes….. No….. | Delay in rainfall stopping | Yes….. No….. |
| Stormy rainfall | Yes….. No….. |  |  |

31b. When you spray chemicals on cocoa farms, do you do the following?

| Spray against the wind | Yes….. No….. | Wear protective boot and overall | Yes….. No….. |
| --- | --- | --- | --- |
| Wear hand gloves | Yes….. No….. | Pour or spray chemical in streams | Yes….. No….. |
| Wear spectacles | Yes….. No….. | Get to taste the chemical | Yes….. No….. |

32. What are the impact mitigation options that you have adopted?

| Adaptation methods | Response |
| --- | --- |
| Diversify more into other crops | Yes….. No….. |
| Diversify into non-farm activities | Yes….. No….. |
| Invest in cocoa drying machine | Yes….. No….. |
| Monitor weather change by indigenous knowledge | Yes….. No….. |
| Re-spray cocoa | Yes….. No….. |
| Reduce time interval for cocoa spraying | Yes….. No….. |
| Irrigation | Yes….. No….. |
| Monitor weather change through the media | Yes….. No….. |
| Planting of hybrid seeds | Yes….. No….. |
| Regular cocoa spraying | Yes….. No….. |
| Change planting and harvesting time | Yes….. No….. |

33. Please, highlight the indigenous knowledge you use to cope with climate change.

1. …………………………………………………………………………………………………
2. …………………………………………………………………………………………………
3. …………………………………………………………………………………………………
4. ………………………………………………………………………………………………….

34. What other coping methods are you using against climate change?

| Method | Response |
| --- | --- |
| Involve in casual labours | Yes….. No….. |
| Withdraw children from schools | Yes….. No….. |
| Reduce household food intakes | Yes….. No….. |
| Beg for food from friends | Yes….. No….. |
| Borrow money for friends to meet family needs | Yes….. No….. |
| Migrate from the village to another village | Yes….. No….. |
| Migrate from the village to another city | Yes….. No….. |
| Take more alcohol | Yes….. No….. |
| Smoke more cigarette | Yes….. No….. |
| Burn fire at home | Yes….. No….. |

35. How do you rank the following efforts to address climate change?

| Adaptation methods | Effective | Non-effective | Not existing |
| --- | --- | --- | --- |
| Collective efforts by all the villagers |  |  |  |
| Individual efforts |  |  |  |
| Local/state motivated efforts |  |  |  |
| Collective weather monitoring |  |  |  |
| Group cocoa spraying |  |  |  |
| Irrigation |  |  |  |
| Monitor weather through the media |  |  |  |
| Credit support from friends and relatives |  |  |  |

**Thank you very much!**
